# Intellectual Property Rights and Access in Crisis

**DOI:** 10.1007/s40319-021-01041-1

**Published:** 2021-03-09

**Authors:** Karen Walsh, Andrea Wallace, Mathilde Pavis, Natalie Olszowy, James Griffin, Naomi Hawkins

**Affiliations:** SCuLE Centre, University of Exeter, Exeter, UK

**Keywords:** Patent, Copyright, Public interest, Access, Open innovation, Open access, Public health, Educational and cultural engagement

## Abstract

The importance of access to intellectual property rights (IPR) protected subject-matter in two crucial areas – public health, and educational and cultural engagement – has been extensively demonstrated during the COVID-19 pandemic. Although they involve separate legal areas, patent and copyright, the common thread linking the two is intellectual property's difficult relationship with access in the public interest. This paper examines the tensions caused by access barriers, the tools used to reduce them and their effectiveness. It is clear that the access barriers magnified by COVID-19 are not restricted to narrow or specific contexts but are widespread. They are created by, and are a feature of, our existing IPR frameworks. Open movements provide limited remedies because they are not designed to, nor can adequately address the wide range of access barriers necessary to promote the public interest. Existing legislative mechanisms designed to remove access barriers similarly fail to effectively remedy access needs. These existing options are premised on the assumption that there is a singular “public” motivated by homogenous “interests”, which fails to reflect the plurality and cross-border reality of the public(s) interest(s) underpinning the welfare goals of IPR. We conclude that a systemic re-evaluation is required and call for positive and equitable legal measures protective of the public(s) interest(s) to be built within IPR frameworks that also address non-IPR barriers. The current pandemic and development of a “new normal” provides a crucial opportunity to comprehensively consider the public(s) interest(s), not just during a global health crisis, but on an ongoing basis.

## Introduction

The COVID-19 pandemic has re-emphasised the importance of access to intellectual property during a crisis. This is especially true in two key areas: public health, and educational and cultural engagement. Although this involves two distinct areas of intellectual property rights (IPR) with differing access barriers – patent and copyright – the common underlying link is their complex relationship with access in the public interest.

A considerable amount has been written on the role of IPR during the current pandemic.^1^ Much of the focus has been on how we could and should use open movements and pre-existing public interest mechanisms to provide access to IPR-protected subject-matter. While we also consider these, it is important to situate access barriers in their broader context. In doing so, we see that the IPR barriers faced during the current pandemic are reflective of wider and pre-existing access barriers.

This paper examines the tensions caused by access barriers, the tools developed to reduce them in the public interest, and their effectiveness. In Sections 2 and 3 respectively, we dissect the elusive terminology of access, public and open, and outline access barriers to IPR-protected subject-matter, questioning whether open movements may sufficiently resolve them. Access is mediated by both IPR and non-IPR barriers. The level of access achieved differs depending on the mechanism used and the public that requires it. Rather than a simple concept, the “public interest” is composed of distinct and differentiated identities and needs. We therefore deconstruct the public interest and explore the overlapping, porous and sometimes competing aspects of the publics and their interests. We make a careful effort to frame the public(s) interest(s) via plurality, the importance of which is explored below. In essence, this terminology reflects our argument that IPR must accommodate a plural public interest if it is to achieve its public welfare goals.^2^

The urgency of access to valuable subject-matter in service of public interests becomes more acute during a public health crisis. Open movements are often advanced as the best solution to reduce access barriers erected by IPRs. For example, in response to COVID-19, numerous calls have been issued for owners of IPRs to grant access to relevant protected subject-matter through open movements.^3^ Such IPR management strategies seek to promote access while centring the public interest within open frameworks. These movements address a pressing and long-standing issue in IPR frameworks – the absence of effective positive measures serving the public(s) interest(s). Despite their clear benefits, we argue that both patent and copyright open movements provide limited solutions because they are not designed to, nor can, adequately address the range of access barriers to promote the public(s) interest(s).

In Section 4, we examine pre-existing legal doctrines as an alternative and additive solution to identified access barriers. In both patent and copyright law, various mechanisms exist to remove access barriers in the public interest. We argue that although these doctrines could be useful, they similarly fail to effectively remedy access needs.

Overall, we find that neither approach, nor their combination, offers an adequate response to reducing access barriers in the public(s) interest(s). Therefore, we recommend that a systemic re-evaluation is necessary. The current pandemic and development of a “new normal” presents a crucial moment to do so. Governments, industry and policymakers have shown their willingness to collaborate. We need to see this as an opening to go further than the immediate pandemic and design new governance approaches that holistically address the range of barriers that compromise access. Left unchecked, these barriers will continue to produce hierarchies among the public(s) and undermine the claims that open movements operate to democratise information, consumption and knowledge production.

We conclude by stressing the pressing need to create positive legal measures protective of the public(s) interest(s) *within* IPR frameworks that also address non-IPR barriers. This requires expressly referencing or defining the public(s)’ rights to access within IPR doctrine, whether based in legislation or jurisprudence. To do so, we must revisit normative understanding(s) of the public interest and how the public(s) may be more effectively served during and outside times of crises.

## Access to Protected Subject-Matter

When evaluating mechanisms protecting the public interest, key terms such as “access”, “public” and “open” remain elusive concepts, often informed or dependent on the respective various viewpoints, disciplines and contexts. Below, we outline the competing understandings of “access” and “public,” before moving on to “open” in Section 3.

### What Is Access?

To begin, access to subject-matter can be impacted by both IPR and non-IPR parameters. These parameters naturally shape which public(s) are able to access and re-use subject-matter, or not.

Indeed, IPRs are premised upon restricting access to subject-matter. This allows innovators and creators (hereafter collectively referred to as “makers”^4^) to limit and leverage access to subject-matter during the relevant period and subject to the conditions of protection. Leveraging access often comes in the form of payment to the rightsholder when the subject-matter is valued enough to be in demand. The opportunity to leverage access is designed to incentivise makers to produce valuable subject-matter, in theory.^5^ This *short-term* restriction is thought to best serve the *long-term* interest of the public (i.e. society or the community-at-large) on the basis that more subject-matter will be created and disseminated as a result.^6^ The short-term public interest is served by providing narrow limitations to that bundle of exclusive rights. IPR rationale thus relies on balancing access with short-term restrictions to generate long-term benefits in the public interest. This system depends on reasonable pricing of protected subject-matter.^7^

Open movements are meant to remove, reduce or modify prohibitive IPR parameters. However, open movements are not generally designed within or by the same regulatory systems that have shaped the IPR regime.^8^ Indeed, they have emerged as a grassroots response to inflexible regulatory and statutory frameworks. Open movements are thus better framed as IPR private ordering strategies because they are meant to map onto, but develop outside of, the regulatory framework of IPR.

Within these strategies, there are different levels or types of access to IPR-protected subject-matter. Practically, a distinction can be made between access to physical materials versus access to their digital formats, particularly with copyright. While these are not entirely separate, they reveal a tension between the physical and digital materials, even where IPR access has been secured. For example, purchasing a physical copy of an in-copyright book will rarely extend access to its digital format. With pandemic related restrictions, the lack of access to *physical* materials, education sites, libraries, and heritage institutions exacerbated the public’s need for *digital* access, as well as the creation of new digital materials to sustain connectivity. IPR frameworks that treat such materials differently require multiple licences to access identical materials published in different formats.

Another level of access refers to permissions granted to the end-user in relation to the physical or digital materials. Free access (or *gratis*) extends access to IPR-protected subject-matter to the public free of charge, often through digital means.^9^ Re-use access (or *libre*) goes further by removing most or all IPR parameters through a licence or public domain dedication in addition to providing free (or *gratis*) access. For example, many cultural institutions extend *gratis* access to platforms for online collections. However, the majority restrict re-use of those images through claims to copyright even where the underlying work is in the public domain.^10^

The last level of access we envisage highlights another difference between copyright and patent protection. The copyright consumer does not necessarily need access to the entire protected subject-matter (the work) in the same way the patent consumer might (the invention). Instead, digital access to view or re-use portions of the works may be sufficient for the types of subject-matter protected by copyright. Certain re-use may already be permitted by statutory exceptions and limitations. By contrast, subject-matter protected by patents cannot be as easily consumed in portions.

Access is also mediated by non-IPR parameters. These include, for example, access to the technologies necessary to view or download the subject-matter (e.g. hardware, software, internet access), whether openly-licensed or with all rights reserved. Accordingly, non-IPR barriers can prevent access to subject-matter made publicly available as both *gratis* and *libre*. With patents, an open invention removes IPR barriers by allowing access to the disclosure, but that does not mean that the invention can be easily produced due to non-IPR barriers, such as know-how. Such non-IPR barriers can impact *who* is able to access, re-use and innovate around open or IPR-protected materials during crises. This ultimately impacts the wider public(s) interest(s), as discussed below.

### Who Are the Public(s)?

Discussing access in the public interest raises questions of who needs access, or in other words, who is the public. Depending on the context, there may be various public*s* and interest*s* to consider.

In simple terms, IPR theory envisions two traditional types of public. The first is the “wider” public, understood as society or the community-at-large residing in a given jurisdiction. As with any legislation, IPR is implemented to serve the wider public in principle. The second public refers to would be users or consumers of subject-matter. They can be thought of as the “immediate” public, who are often the most affected by short-term access restrictions and incur leveraging fees.

The traditional distinction drawn between makers on the one hand (meaning the owners of IPR-protected subject-matter) and the (wider or immediate) public*s* on the other hand is imperfect and overbroad. This is because both groups represent diverse composites that may overlap in practice.

First, makers include inventors and authors, as defined by patent and copyright law. These laws recognise a wide range of rightsholders across extremely varied and specialist fields, from software and hardware engineers, to biotechnologists and geneticists, to artists and performers producing a range of media. Finer distinctions can be drawn among large companies, small and medium-sized enterprises, and individual innovators, with each group facing different market realities.

Second, there are the wider and immediate public*s* in need of IPR-protected subject-matter. However, these categories can be broken down even further according to the relevant jurisdiction due to IPR’s territorial nature, the relevant community, and considerations of inequity (both via the wealth gap and development divide). For example, depending on the country in question, different diseases are prevalent, and treatments may or may not be developed, produced, available or affordable. Accordingly, access to medicines, treatments, devices, diagnostics and testing requires different approaches for the public(s) of low/middle-income countries/communities as opposed to high-income.^11^ The same applies to copyright protected subject-matter, like research, educational materials and accessible format copies, for the public(s) indefinitely homebound, although this is not discussed as widely.^12^ Moreover, the concept of the “public” and its embedded gendered, classist, racial, and geographic associations must be considered.^13^ Consequently, the interest*s* of the relevant public*s* will depend on the type of IPR-protected subject-matter, location, resources and other factors.

Traditionally, makers and publics have been treated separately. These groups are more porous in reality: makers consume subject-matter to produce subject-matter.^14^ Consequently, binary distinctions and generalised framings are inappropriate to reflect the diverse public(s) interest(s) at play in IPR theory and rationale.

### What Are the Access Barriers?

A more complex public reveals nuanced access tensions caused by IPR and non-IPR barriers. Within IPR, the absence of positive measures serving the public interest (in the singular traditional meaning) is better understood when viewed in the context of the IPR balancing exercise between makers’ rights and publics’ rights.^15^ In this paper, we focus on the IPR issues to the extent that is possible. Nevertheless, competing and additive non-IPR barriers in areas like contract and competition law are connected, and might frustrate, overlap, or prevent access for the reasons previously discussed.

In the patent law context, access is restricted to protected inventions.^16^ However, even if patent rights are waived or licensed, re-producing the invention requires the necessary technology (which is also sometimes patent protected), manufacturing capacity and know-how.^17^ These excess limitations are even more pronounced for public(s) in low/middle-income countries and communities due to the development divide and the wealth gap. Other barriers also play a significant role in restricting access, such as funding, regulation, pricing and business models, and distribution.^18^

In the copyright context, access is restricted to a range of relevant subject-matter, like medical-related research and data, as well as educational materials and cultural content. Only the rightsholder may determine whether access will be extended beyond the exceptions and limitations of a given territory. Similar to patents, releasing copyright materials under limited use or open licences allows only the public(s) with technological access to engage via copies. Other barriers can include high pricing and inaccessible business models, publishing models and paywalls, contractual frameworks of platforms and IP overreach, digital rights management tools (DRMs) and digital locks, and distribution.^19^

Although the balance between makers and publics is core to IPR’s premise and rationale, direct references to the wider or immediate publics are largely absent from statutory frameworks. Indeed, these frameworks are dominated by makers’ rights and frame the rights of the public(s) as exceptions to protection or permitted acts. The result is that such “rights” are framed as a defence to infringement, which imposes a burden that is often higher than the one required of makers to secure or enforce protection.^20^ It is not the purpose of this article to give a detailed account of how IPRs favour makers’ interests over public(s) interest(s). This is already well-documented.^21^ We only note this *im*balance, as it is critical when assessing the role and place of open movements in *re*balancing IPR frameworks.

Lastly, distributional inequities raise IPR and non-IPR access barriers. As outlined by Margaret Chon, this is especially true in relation to defining and balancing a more comprehensive understanding of the public(s) interest(s) against IPR’s short-term goals of leveraging access, including who receives or is able to access materials.^22^ Chon has argued the focus on rudimentary high-level measurements of economic welfare reveals growing asymmetries and negative impacts on “development”, capacity-building and social welfare concerns built into international measures such as the Agreement on Trade-Related Aspects of Intellectual Property Rights (TRIPS).^23^ These access barriers are not only concerning in times of *global* crises. Persistent health issues affecting millions of people in low/middle-income countries and communities are not addressed in the same way – there is no scramble for a cure to a disease for which profit margins would be low. There is less attention on the development of cures, let alone access.^24^ Without a more effective means of embedding the public(s) interest(s) in the global IPR system, such inequalities of access to effective diagnosis and treatment will continue.

Access hinges on various technical, financial and other practical resources in the first place.^25^ The application of each given filter reduces access to an increasingly narrow, singular and privileged public. In effect, these filters produce hierarchies among the public(s) and undermine the claims that open movements operate to democratise information, consumption and knowledge production.^26^ This is crucial to consider against the necessary infrastructure and digital technologies for participation, as well as when designing more equitable approaches within the global IPR regime in the public(s) interest(s).

## Open Movements – A Panacea?

Open movements are often proposed as *the* solution to IPR barriers. Indeed, during the COVID-19 pandemic, there have been numerous calls to use open movements and for government intervention around IPR access.^27^ In the IPR context, open movements augment existing frameworks and allow access to materials that would normally be restricted. Yet, “open” plays out differently in patent and copyright law. As will be elaborated on below, open innovation (OI) is more commonly used in relation to inventions, whereas open access (OA) has a long-standing attachment to copyright law.

Interestingly, funding approaches also differ in the patent and copyright contexts, which can impact “open” obligations. With patents, the makers of both privately and publicly funded initiatives retain the capacity to apply for IPR. For privately funded innovations, this complements the underlying IPR rationale. However, when public funding is involved and the maker retains IPR, this allows the rightsholder to charge the public for that invention again, with no restriction on cost. There have been numerous calls for public funding bodies to insert IPR or pricing conditions into funding obligations.^28^ Funders increasingly recognise the need to ensure the public welfare benefit of the research that they fund, but significant progress remains to be made.^29^

With copyright, public and private funding schemes often set OA obligations to release research and data under open licences or tools.^30^ For example, the Bill & Melinda Gates Foundation, Ford Foundation, and William and Flora Hewlett Foundation require materials to be made available via a Creative Commons CC BY licence.^31^ In 2018, a group of 11 funders called cOAlitionS announced they will require funded research to be published in OA journals, platforms, and repositories without embargo.^32^ Indeed, funders have been instrumental in changing scholarly publishing practices in relation to the open sharing of data, research, and publications as a condition of funding.^33^

Against this backdrop, it is worth examining open movements to determine whether grassroots and industry driven initiatives can sufficiently fill the gaps left open by regulatory and statutory frameworks and promote the public(s) interest(s).

### Open Movements in Patents for Public Health

In patent law, OI is becoming more popular. Developments through OI include Mozilla Firefox and Moodle, and this type of innovation has been used by companies such as LEGO and NASA.^34^

#### Open Innovation Theory

OI has been mostly defined in reference to its more common and well-known counterpart – closed innovation.^35^ Closed innovation involves research taking place within a particular company. Inventions are generally protected by patents (if granted) with access completely controlled by the rightsholder. OI has been defined as “the use of purposive inflows and outflows of knowledge to accelerate internal innovation and expand the markets for external use of innovation respectively”.^36^ Companies that adopt OI use both internal and external knowledge to develop new inventions, often in partnership with other firms. In doing so, they rely on private ordering, for example, using contracts to construct terms of access to IPR. Many of these initiatives have increased information flow, resulting in quicker development compared to closed innovation models. However, OI often remains for-profit and restricted by patents. As a result, how and when licences are granted depend on the rightsholders.

Geertrui Van Overwalle has distinguished this type of “firm-centred open innovation” (FCOI) from “community-centred open innovation” (CCOI).^37^ While the results of FCOI are commonly restricted, CCOI has broader parameters. It involves a group of makers connected by “shared worldviews, norms and identities”.^38^ Accordingly, there are different motivations to innovate than with traditional IPR incentive arguments. The resulting inventions are generally not patent protected, not-for-profit, and made more accessible to wider and immediate public(s) than inventions produced under traditional innovation models.^39^ Van Overwalle distinguishes both groups from individual user innovators, whose contributions could be their own projects, or a part of group or community innovation.^40^

#### Open Innovation in Practice

OI often takes the form of patent pools and pledges. There are a number of examples of rightsholders limiting their IPR through conditional mechanisms. For example, the UN Medicines Patent Pool (MPP) negotiates licences with rightsholders to provide access to medicines to low/middle-income countries.^41^ While such initiatives can serve to address IPR-related access barriers, some can also raise competition concerns and entrench the power of existing market incumbents with strong patent portfolios, disadvantaging both wider and immediate public(s).^42^

Open source software can be considered a subset of OI, falling under either CCOI or individual user innovation.^43^ Software can be patent protected in the traditional sense and thus fall within OI.^44^ Alternatively, open source licences can permit the use and re-use of software. This allows others to view, share and adapt the software so long as any modifications are also shared under the same permissions. Open source can go beyond OI by implementing licences that ensure users retain access to downstream innovations.

During the COVID-19 pandemic, there has been a scramble to develop treatments and vaccines to counter this disease alongside the parallel scramble by IP scholars, activists and practitioners to enable access to these innovations. The specific response thus far has resulted in two group initiatives being set up to grant access to patented inventions and technological know-how.

The first initiative is the World Health Organization (WHO) COVID-19 Technology Access Pool (C-TAP). On 29 May 2020, Costa Rica and the WHO launched a voluntary IPR pool for COVID-19 related technologies. The Pool compiles all pledges made under the WHO’s call to action for sharing COVID-19 “health technology related knowledge, intellectual property and data” and draws on relevant data from pre-existing pools.^45^ C-TAP is supported by Unitaid and MPP.^46^ As such, C-TAP could increase the availability and exchange of information needed to respond to the pandemic and “would make country-by-country, product-by-product confrontations over such knowledge redundant.”^47^

The second initiative, developed by the Open COVID Coalition, is the Open COVID Pledge. Led by legal and scientific academics and practitioners, this pledge “calls on organisations around the world to make their patents and copyrights freely available in the fight against the COVID-19 pandemic.”^48^ It is another mechanism that can be used to fulfil the WHO’s call to action. By signing the Pledge, rightsholders commit to openly-license medical equipment, testing kits, software, AI and biotech solutions to help fight the virus through a pre-prepared and very simple licence.^49^ The pledge currently applies to more than 250,000 patents worldwide.^50^ Generally, the pledge accepts three categories of licences: standard, compatible and alternative.^51^ The most commonly used standard Open COVID License (OCL-PC v1.0) grants a “non-exclusive, royalty-free, worldwide, fully paid-up license (without the right to sublicense)” for products, services and processes for the sole purpose of ending the COVID-19 pandemic.^52^ The OCL is retroactive to December 2019 and expires one year after the WHO announces the end of the pandemic (or 1 January 2023, unless extended by the rightsholder).

There have also been a number of individual voluntary actions taken to allow access to certain medicines and technologies relating to COVID-19.^53^

#### Positives

There are important advantages to FCOI for firms adopting this model. Inventions are developed in collaboration. The eventual rightsholder is rewarded through licence fees, but licences are granted on fair terms to the firms involved and can therefore be used and developed more widely. This model mostly depends on the rightsholder. If their invention is not deemed to be a standard essential patent, they have a choice whether or not to licence voluntarily and set charges.^54^ Access is restricted by IPR protection (and non-IPR barriers), similar to the closed innovation model, which impacts both the public(s) and re-users.

With other types of OI, for example, open source software, the source code is made accessible to all and it can be developed, improved and redistributed under the condition that any downstream innovations must also be open source. This innovation can be community-centred or individual, allowing for more re-users and public(s) to be considered, to some extent. Living labs are an example of an OI model that enables users to be more actively involved in the innovation process.^55^

Although access remains somewhat restricted in OI given the continued use of IPR and/or the requirement of a licence to re-use subject-matter, this is unavoidable. Reducing all IPR-protection could result in downstream re-use that makes even minor improvements being re-subjected to IPR-protection. Power would shift to the new IPR owner, who would control the re-use of that improvement. This would also raise new IPR access barriers for all public(s) as the new owner would also control the development of further improvements.

These apparent benefits have led to the introduction of the above initiatives to assist with the COVID-19 pandemic. Because these pools and pledges can be fast-moving, individually tailored, and remove barriers to information, they can adapt to the pandemic in ways regulatory frameworks cannot. However, the ultimate impact of these responses upon access to vaccines, treatments and other inventions relevant to COVID-19 is yet to be seen.

#### Concerns

Despite the benefits of FCOI, the resulting invention remains in-house and participants are chosen by the firm. CCOI shows significant promise; however, a question remains as to whether this type of OI is more suitable to certain industries where research costs are lower, for example, in computer software. Furthermore, although OI may extend access beyond what traditional IPR frameworks achieve, access is not guaranteed to all public(s). Individuals, communities and companies without the technological or manufacturing capacity and know-how will not be in a position to build upon these innovations.

With both FCOI and CCOI, access barriers remain. For the firm-centred approach, inventions remain restricted by patent protection and restrictive licence agreements. For the community-centred approach, inventions remain restricted by barriers related to the wealth gap.

Both the C-TAP and Open COVID Pledge are promising initiatives. Temporarily relinquishing IPR barriers could lead to more effective responses to the pandemic. However, the effectiveness of the Pool and Pledge will entirely depend on the pledgors and what they decide to pledge. Multiple countries, intergovernmental organisations and nongovernmental organisations have supported the WHO’s call for action, and an impressive line-up of technology, information and communication companies like Intel, IBM, and Microsoft have signed on.^56^ Such broad and wide-ranging support is encouraging. However, most pharmaceutical companies have yet to sign on.^57^ In the absence of key players’ support, the impact of these initiatives on the wider availability of COVID-19 pharmaceutical treatments and vaccines will be minimal. Such initiatives run the risk of being used as a mere public relations exercise. When entities with little IP in relevant fields or little potential profit to lose become pledgors, but those who hold key patents remain outside the initiative, the real-world impact is lessened.

These ad-hoc responses allow for flexibility but require further consideration to ensure responses are equitable and sustainable. Ellen ‘t Hoen questions how key technologies will be made available to the public(s) and stresses the “urgent need for global collaboration, access conditions on public funding and upfront agreements on access and affordability with those engaged in the development of new medical technologies.”^58^ Of note, is that the majority of funding for COVID-19 research and innovation has been provided by government and charitable organisations. However, EU officials have stated that “pharmaceutical companies who will receive the funding will not be requested to forgo their intellectual property rights on the new vaccine and treatments, but they should commit to make them available worldwide at affordable prices.”^59^ These requests for access at affordable prices are rarely followed up by enforceable conditions in funding however, and what is “affordable” is also not clearly specified, nor tailored to the realities of individual public(s).

The next step will be to ensure that all public(s) receive treatment in a fair and equitable manner. As of 8 July 2020, €15.9 billion has been raised by the Coronavirus Global Response Initiative for universal access to tests, treatments and vaccines, and this fund will go some way to providing for access.^60^ The WHO (supported by organisations including the Bill & Melinda Gates Foundation, Gavi and CEPI) have also launched the Access to COVID-19 Tools (ACT) Accelerator to assist with development and equitable access to tests, treatments and vaccines.^61^ The COVAX pillar is implementing a pooled procurement and equitable distribution mechanism for vaccines.^62^ There is great political will to sustain public funding of treatment costs. Moreover, the size of the market for COVID-19 vaccines and treatments is likely to be so large that companies will be able to recover vast sums even if there is a tiny return on each individual item. This could make the pricing of individual items lower than for vaccines and treatments for other diseases. However, the overall cost of providing global access to vaccination will be enormous.

For high-income countries, access agreements to treatments and vaccines have been and will be relatively easily reached. The US acquired the remaining doses of remdesivir, a key treatment for COVID-19 in June 2020.^63^ The US and UK entered into advanced purchase orders for vaccines which are currently being rolled out, prompting discussions on vaccine nationalism.^64^ However, whether all public(s) in high-income countries will have equal access to the vaccine will depend on how individual governments finance the roll out.

Further, for public(s) in low/middle-income countries, access barriers such as availability and affordability will likely endure.^65^ The vaccine is currently being administered across the world. However, at the time of writing, out of the 42 countries administering the vaccine, only six are middle-income countries; the vaccine has not been dispensed in any low-income country.^66^ A request to waive key provisions of TRIPS and for the global sharing of technology and know-how was made by India and South Africa to the WTO.^67^ If accepted it would allow a circumvention of key IPR and non-IPR access barriers. This would be especially important for low/middle-income countries. However, and despite the urgency of this situation, a decision has been delayed until 2021.^68^

Pandemics are not the norm, and global access to treatments and vaccines for orphan diseases or diseases less common in high-income countries remain problematic. As we argue below, lessons learnt from the COVID-19 push for access must be applied to wider contexts.

### Open Movements in Copyright for Education and Cultural Materials

Differences in patent and copyright entitlements naturally lead to differences in their respective open movements. As copyright arises automatically once an original work is expressed in a material form, individuals have more power to determine subsistence and access parameters.

#### Open Access Theory

In copyright law, the term “open access” is used broadly and inconsistently across science, culture, education, publishing, and technology fields. One reason these disparate understandings have emerged is due to scholarly publishing practices. This has particular consequences for scholarly research across all fields, given the publishing requirements in academia and size of the market. Publishing culture frames the act of making information viewable without a paywall or fee as satisfying OA standards. In this way, open is widely presented as synonymous with *gratis* access, with some models extending additional permissions for user activity already protected by copyright law, such as educational use.

By contrast, Peter Suber distinguishes between non-IPR and IPR access barriers via the terms *gratis* and *libre*.^69^ As discussed in section 2, *gratis* access signals free of charge (non-IPR), while *libre* access is both free of charge and free of most copyright and licensing restrictions (non-IPR and IPR). “Open” access thus requires both *gratis* and *libre* access.^70^ This aligns with international OA initiatives that qualify materials according to their commercial reusability.^71^ Under such initiatives, only materials published under tools and licences that permit commercial re-use are open compliant.^72^ This has an impact on re-users as only materials with *libre* access can be re-used without risk. However, not all re-users will benefit as other non-IPR barriers remain.

*Gratis* access is important to consider on its own when IPR does not arise. For example, basic research data, descriptive data, and faithful reproductions of public domain works may not qualify for copyright protection when the output does not satisfy the relevant threshold for protection. In theory, this eliminates the need for setting *libre* access parameters, while increasing the focus on *gratis* access via a commitment to voluntarily make the non-original materials publicly available for access and re-use.

OA extends to any original subject-matter that a copyright might apply to, such as: software (open source), data (open data) and other cultural and educational subject-matter (open educational resources). However, the inconsistent use of “open” introduces legal uncertainty in practice.

#### Open Access in Practice

Any elimination of a non-IPR barrier (e.g. paywall or subscription fee) to IPR-protected materials is commonly presented as “open” in and of itself. Within scholarly publishing, this has been driven by journal and repository practices via the adoption of green OA (i.e. the author deposits the article with repository or self-archives the work) and gold OA (the final version of the article is made available upon publication).^73^ These delivery models are primarily *gratis* based, but some extend *libre* access.

During COVID-19, the general response has been to temporarily adopt limited *gratis* access models. More than 30 leading publishers, such as Oxford University Press, Elsevier, Wiley and Springer removed subscription-based restrictions for COVID-19 research, textbooks and other educational materials.^74^ In addition, various subscription-based media publishers, such as the New York Times, Bloomberg, The Atlantic and Publico did the same for COVID-19 articles. In other cases, licensing organisations temporarily revised their educational licences to allow for higher copying thresholds.^75^

At the same time, organisations, funders and grassroots initiatives were able to apply pressure to closed publishing models, sometimes by circumventing them entirely. One example includes a call from the National Science and Technology Advisers of a dozen countries for publishers to make COVID-19 research, and the supporting data, available through PubMed Central.^76^ The Wellcome Trust issued a similar commitment and call for researchers, journals and funders, with more than 140 signatories.^77^ A letter to WIPO “to take a clear stand in favour of ensuring that intellectual property regimes are a support, and not a hindrance, to efforts to tackle both the Coronavirus outbreak and its consequences” was signed by 32.5 million educators, 2.5 million libraries and archives, 45,000 museums, and 200 copyright scholars across 199 countries.^78^ Other examples of public(s) support for reduced barriers include statements by copyright specialists to support maximising copyright limitations and exceptions by interpreting them to provide the necessary flexibility for online education in the current crisis,^79^ as well as individual researchers widely publishing materials prior to (lengthy) peer review processes via platforms like SSRN, bioRxiv and Gisaid.^80^

Galleries, Libraries, Archives and Museums (GLAMs) have taken similar blended approaches to OA. While some GLAMs release all non-original data *gratis* and *libre* (e.g. digital surrogates of public domain works, collections data, metadata, paradata, etc.) via open tools, others claim copyright and release data via open licences (CC BY, CC BY-SA).^81^ The majority of GLAMs with digital collections online make them viewable *gratis* while reserving all rights through an (alleged) copyright claim or by releasing data via closed licences (e.g. Creative Commons NC and ND).^82^ Data may be released *gratis* and/or *libre* via institutional websites, while platforms such as Wikimedia Commons and GitHub set *libre* conditions for any data uploads.

COVID-19 has so far had no measurable impact on GLAM OA policies. Nor has it stimulated any OA adoptions by non-open GLAM institutions despite the public(s) inability to physically access collections. This is likely because OA transitions are time and resource intensive, even under normal conditions. During indefinite closures, with staff working from home, priorities have shifted to cost reduction, increased digital engagement through social media, and digital collections management.^83^ The current situation has exacerbated the digital divide for institutions without digital resources, expertise and presence.^84^

OA adoptions may pause due to (misplaced) desires to retain commercial licensing benefits and generate revenue through online activities. Studies by UNESCO, ICOM and NEMO have detailed the dire economic impact of COVID-19 on an already financially-stressed sector.^85^ At least one national museum has recalled its public domain policy in recent months, though reasons have been attributed to the general desire to license images again rather than as a direct response to COVID-19.^86^ However, research shows the economic inefficiencies of maintaining licensing schemes, which often operate at a cost to cultural heritage institutions.^87^

Lastly, Open Educational Resources (OER) initiatives across multiple fields and sectors have been designed with OA in mind. In November 2019, UNESCO adopted the UNESCO OER Recommendation as the first and only international standard-setting framework worldwide. The recommendation defines OER as learning, teaching and research materials that are marked as public domain or released under open-compliant licences that permit no-cost access, re-use, re-purpose, adaptation and redistribution (i.e. *gratis* and *libre* access).^88^ This benefits many wider and immediate public(s) and re-users who can distribute OER materials to digitally-isolated publics.

GLAMs and OER have played an important role in home-schooling, distance learning, and public(s) engagement. Many GLAMs have hosted virtual exhibitions and lockdown tours, turning to social media to publish materials, engage audiences and participate in the #MuseumsFromHome initiative.^89^ While this results in significant amounts of new IPR, there have been no corresponding pledges to release GLAM-generated IPR via open licences or tools for COVID-19 access, even temporarily.^90^ By contrast, UNESCO launched the COVID-19 Global Education Coalition and issued a call to the global community via an open letter “to support the use of OER for sharing learning and knowledge openly worldwide, with a view to building more inclusive, sustainable and resilient Knowledge Societies.”^91^ Indeed, UNESCO is supporting a number of OER initiatives in relation to COVID-19 to reduce IPR and non-IPR access barriers to a range of relevant materials, including for digitally-isolated publics.^92^

#### Positives

One advantage of copyright is that all or select rights in the bundle may be waived (or at least not enforced). This has allowed open movements, like Creative Commons (CC), open GLAM and OER, to develop innovative IPR strategies to confront ongoing problems around the lack of cross-border exceptions and limitations for re-use, as well as the lack of legal clarity and consistency around originality standards.^93^ For example, open licences and tools are legally interoperable, machine readable, and international. CC has even revised its tools to better convey the limitations of a public domain status and the express intention of enabling cross-border re-use: these include the Public Domain Mark 1.0 and the CC0 1.0 Universal Public Domain Dedication.

As implied, the CC0 tool is premised on a waiver, and thus the existence of a copyright. Technically, CC0 is therefore inappropriate for materials that fail to satisfy the originality standard. However, the tool’s legal code expressly considers users as central to the Public Domain Dedication relationship.^94^ Although no rights may arise in the jurisdiction of origin (e.g. moral rights in the United States, which are very limited in scope), other jurisdictions *might* recognise rights in the materials (e.g. moral rights in France, which are broad and perpetual). In this sense, the Public Domain Mark is imperfect, because it is territorial to and dependent upon the public domain law in the jurisdiction of origin, which can differ from the re-use jurisdiction.^95^ The CC0 1.0 Universal Public Domain Dedication thus provides cross-border re-users with legal certainty that even if rights arise, the rightsholder will refrain from enforcing them. The design of this IPR strategy accounts for different cross-border publics. Moreover, open licences enable rightsholders to reduce access barriers to some rights in the bundle while retaining others, such as CC BY (Attribution) and CC BY-SA (Attribution-ShareAlike). These licences are irrevocable, which provides greater legal certainty to downstream makers re-using IPR-protected materials in cross-border environments.

Open compliant materials (i.e. Public Domain, CC0, CC BY, CC BY-SA, etc) can be uploaded to platforms like Wikimedia Commons and Wikidata. Considering the infrastructure required to host online collections, Wikimedia platforms have helped reduce digital access barriers by allowing rightsholders and content providers to directly upload high-quality materials for access and re-use. Indeed, open movements have both fed and benefitted from these and similar platforms. Such websites have been a driver for OA via the upload conditions, providing more open compliant materials to the public for re-use. In return, OA has been a driver for knowledge production via the websites through which the open compliant materials are contributed.^96^ Accordingly, public(s) and maker(s) are comprehensively built into the structure and IPR strategies of Wikimedia platforms like Wikipedia, which consistently falls in the 10 most visited websites in the world.^97^

#### Concerns

Unlike OI, with OA, concerns are particularly related to non-IPR barriers for the immediate public(s). IPR concerns relate to the technical and cross-border nature of digital dissemination.

Practical barriers arise when makers fail to embed digital materials with machine-readable interoperable rights statements. Once divorced from their origins, the open status of the materials will fail to follow their circulation online. Cautious downstream users may forgo re-use at the risk of legal liability, even when materials appear to be non-original (e.g. digital reproductions of public domain works).^98^

Even when rights statements follow the materials, legal barriers can arise. IPR remains jurisdictional. CC licences and their application may change from one jurisdiction to the next depending on differences between moral rights, the term of copyright protection, and other national IPR law parameters. One significant barrier is the question of originality, which is legally required to enforce copyright entitlements. The instantaneous nature of copyright protection enables a system that allows the party who would benefit from an exclusive right to determine whether the work is sufficiently original in the first place. Any conflict or challenge to originality must be litigated in court. Taken together, these conditions produce disincentives to enforcement by the alleged rightsholders.^99^

This occurs when makers claim copyright in non-original subject-matter and restrict access or re-use by reserving all rights or releasing materials through non-open compliant licences. Even when no rights are claimed, *gratis* access may be withheld. No positive or enforceable legal obligations exist to release non-original materials in the public interest.

The development divide and wealth gap raise the most significant access barriers. As noted above, finances are required to secure IPR access. The greater risk is overlooking the structural inequities that prevent both IPR and non-IPR access.^100^ Access, participation and innovation with IPR-protected content in *libre* access, as envisioned by open movements, requires access to the necessary digital technologies, something unrelated to IPR. As a result, the enjoyment of *libre* access is being limited to privileged public(s), now in the position to gain further benefits.^101^ OA cannot resolve these underlying barriers and may deepen the development divide and wealth gap,^102^ especially during crises.^103^

To this point, studies on the impact of COVID-19 on the heritage sector show GLAMs may shutter at an alarming rate. UNESCO estimates more than 10% of museums may never reopen.^104^ In the United States, that number is as high as 30%.^105^ The loss of institutional and individual knowledge will be immeasurable. For those that remain operational, there have been increased attempts to collect cultural engagement during COVID-19 on digital platforms. The growing digital divide means significant portions of the public are not only excluded from these archival collections, but also the knowledge generated around them due to their inability to both contribute and participate.^106^

### Open Movements as a Response to Access Barriers

Releasing IPR-protected materials under open licences and tools, in and of itself, neither results in the public(s) having access, nor satisfies wider public welfare goals as envisioned by IPR.

Open movements in patent and copyright law have clearly addressed a number of issues relating to access barriers. Yet, significant gaps remain for a number of publics. This is mainly due to the voluntary nature of open movements. For this reason, open movements cannot resolve every IPR barrier. This is particularly apparent during times of crises. During COVID-19, impressive initiatives have emerged, but they are participant-dependent and time-limited. The long-term impact of the virus must also be taken into account, which will outlast many of these initiatives. Finally, the existence of life-threatening diseases that affect public(s) in low/middle-income countries and communities and the likelihood of future pandemics and other crises must be considered.

The temporary status of COVID-19 open initiatives brings benefits and drawbacks. One benefit is that their temporary validity may attract participants who would not usually engage in open movements due to the irrevocable status of many tools and licences. Most if not all of the subject-matter being pledged was developed outside of OI strategies, but has now adopted those characteristics. This may incentivise companies to opt for OI in the future, given the mutual benefits to maker(s) and public(s) in terms of innovation efficiency.^107^ However, temporary also means that these initiatives are narrowly defined and less sustainable. For example, as mentioned, the standard OCL expires one year after the end of the pandemic. IPR access barriers therefore remerge upon expiration. Even with non-IPR access, scholarly and media publishers are beginning to reintroduce paywalls to COVID-19 and educational materials.

While open movements are filling gaps in ways the IPR regime is unable, such strategies frame access for maker(s) and public(s) within the relevant materials and IPR area. This results in narrowly tailored IPR strategies for some immediate public(s) without non-IPR considerations for the wider public. Significant room remains to develop more equitable access strategies and obligations that are more appropriately designed and supported by IPR regimes, so long as the concerns noted above are confronted.

Open movements have somewhat addressed IPR access barriers, but a question remains as to why open movements were seen as necessary given the existence of traditional public interest legal doctrines.

## Existing IPR Legal Doctrines – An Alternative or Additional Solution?

Multiple IPR doctrines have been developed to provide access in the traditional public interest, many of which find their basis in the flexibilities of the TRIPS Agreement.^108^ In this section, we consider how these doctrines could be applied in public health crises and evaluate their capacity for reducing access barriers. We approach this section primarily from a UK perspective. Many jurisdictions have similar provisions; thus, this analysis can be applied in broader contexts. However, the territorial nature of these doctrines limits their application in cross-border contexts, which are especially important during crises.

### Patent Law

Patent law provides various mechanisms that aim to take the traditionally conceptualised public interest into account. Although a specific public interest doctrine does not exist in UK patent legislation, the concept is inherent to the patent system. The balance between granting a patent and restricting access to the public has been considered in each requirement for and restriction to patentability.^109^ Patent offices examine the exceptions and exclusions to patentability, users can rely on certain defences to infringement, and governments can impose compulsory licences or approve crown/government use. Courts also have discretion when deciding on many of these issues when raised, including also whether or not to grant an injunction.^110^ In this way, the patent system includes checks and balances whereby the public interest can be considered at multiple points in the life cycle of a patent.^111^

However, because of these inherent inclusions, the public interest rarely receives additional attention. There appears to be a reliance on the fact that patent requirements and restrictions have taken the public interest into account, and therefore, with their application, the public interest has been accommodated. In times of crises, it should be possible to re-consider this balance with public(s) interest(s) in mind and use pre-existing legal mechanisms more readily to allow access. This section considers these possibilities and how useful they are.

#### Exceptions and Enforcement

- Exceptions

Exceptions to patentability prevent rights arising in situations where patents are considered to be against the public interest. There are two relevant sets of exceptions in the public health context: *ordre public* and morality; and medical and diagnostic methods. These provisions are considered during examination and can be raised when opposing the grant of the patent, or in revocation proceedings post grant.

In Europe and the UK, patents are refused for inventions if their commercial exploitation would be contrary to *ordre public* or morality.^112^ The European Patent Convention (EPC) provides no definition for these terms but the Board of Appeal in *Plant Genetic Systems* offered vague working definitions.^113^ Case law suggests the possibility of abuse of the invention is not sufficient to deny patent protection, and if the invention can be exploited in one way that would be considered moral, protection will be granted.^114^ The exception has been applied very narrowly and it is unlikely that it would be stretched to restrict the patenting of a vaccine during a public health crisis. Patents being used to limit or restrict access to a technology that would be useful to the public would not fall under this exception.^115^

The second set of exceptions relevant in the public health context refuses patents on methods for treatment by surgery or therapy, and diagnostic methods.^116^ These exceptions are also narrow in scope. With diagnostic testing, if *in vitro* testing is required, these methods fall outside the exception because tests are carried out on samples removed from the body rather than practiced on the body, as the exception requires.^117^ Given that the vast majority of modern diagnostic tests require some laboratory element, the scope of this exception is so narrow as to be practically irrelevant. Treatment by surgery or therapy practiced on the human body may not be patented, but any devices or drugs used in that therapy fall outside the exclusion. The practical effect of these exceptions in times of public health crisis is therefore limited.

Exceptions to infringement provide a defence against otherwise valid patents in specific circumstances.^118^ One such defence is for acts done for experimental purposes.^119^ An act will fall within the defence if its purpose is to discover something unknown or to test a hypothesis, but it must relate to the subject-matter of the patented invention.^120^ This would be useful during a crisis to identify new uses of patented therapies, pharmaceuticals and products. However, once the new application has been validated and becomes routine, its use will not be covered by the defence.^121^ A licence on the original subject-matter would be required to use the new material, and the maker of the new invention could choose to apply for a patent, thus further restricting access.

Another relevant defence is for the making of a prescribed medicine in a pharmacy.^122^ The practical application of the defence is limited to cases where such preparation is medically necessary rather than economically necessary, as most pharmacists dispense pre-prepared medicines where possible.^123^ In the context of a public health crisis, the defence could become relevant where commercial manufacturing capacity is limited. However, this would only be in the relatively rare situation where hospital pharmacies are capable of the preparation of the relevant drug.

(b) Enforcement

Enforcement is also relevant with regards to the court’s discretion to refuse granting injunctions. Even if a patent is held to be valid and infringed, a court has the discretion to choose the appropriate remedy. Remedies include an injunction, damages or an account of profits, and delivery up or destruction of infringing articles.^124^ For example, if another company makes a patented medicine to provide access, the rightsholder can sue for infringement. During a public health crisis, a court could choose to refuse an injunction and award damages instead.^125^

Although there is little scope for the public interest to be considered when enforcing a valid patent, recent English case law demonstrates a potential, if limited, role for the stay of an injunction on public health grounds.^126^ This would only be possible if there is a lack of viable alternatives for patient access to life-saving treatment. The circumstances in which such a stay may be awarded are rare and damages would likely follow.

#### Non-Voluntary Licensing

- Compulsory licensing

A compulsory licence, granted by the government, compels a patentee to allow use of their patented invention under reasonable terms.^127^ However, there are many onerous requirements to fulfil prior to the authorisation of a compulsory licence. As a result, their effectiveness is limited. For example, an application can only be made three years after a patent has been granted. Applicants must also show that demand for the product is not being met and that attempts have been made to reach an agreement on reasonable terms through a voluntary licence within a reasonable period.^128^ Data exclusivity must also be taken into account in the EU context with regard to pharmaceuticals.^129^

This is not to say that compulsory licences are not useful. The mere existence or the threat of applying for a compulsory licence often forces patentees to come to voluntary agreement or to take alternative action.^130^ A recent example occurred in the UK with medicines for cystic fibrosis. During a three-year negotiation, it was argued that the government should enforce a compulsory licence on these medicines given their cost was unaffordable for the NHS.^131^ With mounting public pressure, a voluntary licence was agreed.^132^Another example can be seen in the COVID-19 context. AbbVie, a biopharmaceutical company, has stated that it will no longer enforce its patent rights worldwide on a medicine used to treat COVID-19 symptoms. However, this was only after a compulsory licence had been issued.^133^

This mechanism is one of the most useful to re-balance the patent bargain in the public interest. However, it places a significant burden on the applicant, especially during a public health crisis. Moreover, it is worth noting that in a non-pandemic situation it took three years of negotiations to reach an agreement on an affordable voluntary licence for cystic fibrosis medicines, which only benefits the public in the UK. For low/middle-income countries, movement is much slower and efforts to provide affordable access to treatment for HIV/AIDS, hepatitis C and tuberculosis remain ongoing.^134^

In response to COVID-19, some countries, such as Germany, France and Chile, have introduced legal measures to make it easier to grant compulsory licences.^135^ In Germany, legislation was introduced relevant to all pandemics, whereas in France and Chile, the emergency laws are COVID-19 specific.^136^ These types of legislative changes could be of great benefit if widely introduced in low/middle-income countries during and beyond the context of the pandemic to include all public health crises, especially considering that many high-income countries have declared themselves ineligible to be importing members under Art. 31^bis^ TRIPS.^137^

(b) Crown Use

Another option is to employ the Crown Use provision whereby any authorised department or person can make, use or sell a patented invention “for the services of the Crown” without the permission of the patentee, so long as it is in the public interest to do so.^138^ Further detail states that “the production or supply of specified drugs and medicines” would be included in services of the Crown.^139^ Finally, there are special provisions relating to Crown use during a declared emergency, which allows use of the invention in many other circumstances, including “for the maintenance of supplies and services essential to the life of the community”.^140^

Crown Use was successfully used as a defence to infringement recently in *IPCom v. Vodafone*.^141^ Attempts were made to limit the provision’s scope, but these were rejected by the court.^142^ The provision was also raised in *Evalve v. Edwards*, where the court gave the example of generic versions of life-saving drugs engaging the public interest in “special cases, such as a novel pandemic disease”.^143^

During a public health crisis, Crown Use could be invoked successfully, if required. Given the legislative and recent jurisprudential applications of Crown Use, the requirements would likely be satisfied. However, one crucial aspect to consider remains. The appropriate royalty to be paid is only negotiated after use and so the eventual cost to the public is unclear at the point of implementation and may prove high. The uncertainty around the cost of invoking Crown Use may result in hesitancy. Even so, it remains an option where negotiations with a patent holder prove intractable.

#### Patent Evaluation

Overall, these legal doctrines are underdeveloped and underused, particularly when considering the plural public. The possibility of the exceptions and enforcement mechanisms reducing access barriers is limited at best. While they may permit a degree of research and development of new therapeutics and vaccines, they will not enable access to those products, except in very limited circumstances. Courts refusing injunctions on public interest grounds would be helpful, but only arise in rare circumstances and rely on judicial discretion.

More promising are the non-voluntary licence mechanisms. Compulsory licences may be effective in reducing access barriers, but in practice, their effectiveness arises primarily in the threat of their use, rather than their implementation. Crown Use seems to be the best fit to permit access to patented diagnostic equipment, medications and other treatments during a public health crisis. However, questions remain relating to payment.

As well as the gaps identified above, especially with regard to licences, these mechanisms share some overarching barriers, as previously mentioned. If a licence is granted, be it voluntary, compulsory, or as a result of Crown Use, there are remaining non-IPR access barriers relating to technology transfer, data and market exclusivity, and know-how, which are necessary to make the invention work. It is important to note that trade secrets may also be operating in the background.^144^

Finally, and most importantly, for equitable access, especially for public(s) in low/middle-income countries, lack of local manufacturing capacity, and inability to import due to trade restrictions can be a significant barrier to access. These points must all be considered and addressed prior to the emergence of a public health crisis so that access is guaranteed for all public(s).

### Copyright Law

Copyright law has various mechanisms that were developed to advance the public interest. The most commonly relied on is fair dealing, found in Chapter III of the UK Copyright, Designs and Patents Act (CDPA) 1988. Fair dealing contains a list of permitted acts which support copying and access in the public interest.^145^

In a crisis, how these acts are framed and extended in the public interest will naturally depend on the act and the context of the crises or emergency situation. For example, with respect to UK educational institutions, it has been argued elsewhere that permitted acts are too narrow for consideration of a pandemic and also that the public interest defence would likely fail but may be relevant to calculating remedies.^146^ Instead, legislative expansion, voluntary collective licensing and alternative models for academic publishing might be used in tandem.^147^ This suggests that legislative reform alone is insufficient to expand existing categories and/or design new permitted acts adequate for responding to crises in the public interest, particularly in a common law system which relies on precedent to inform entitlement boundaries. For these reasons, we consider the existing scope of doctrines outside of fair dealing and whether they provide adequate approaches to responding to public crises. These include the doctrine of public interest and others related to public interest, such as ideas, morality, non-voluntary licensing and restraint of trade.

#### Public Interest

Section 171(3) CDPA states that “nothing in this Part affects any rule of law preventing or restricting the enforcement of copyright, on grounds of public interest or otherwise.” The doctrine has been developed through case law and the discussion has been piecemeal. Furthermore, it is clear that different judges have different views about what the test for public interest should be.^148^

A number of cases have considered the relationship between copyright and the public interest.^149^ In *Lion Laboratories*, O’Connor LJ took a broad approach to whether copyright prevented a work’s publication and re-use, stating that “once the public interest has been properly aroused and brought out in public” the subject-matter “should be before the public and not restrained from use”.^150^ Aldous LJ restricted this framing in *Hyde Park Residence Ltd*, suggesting a court would only consider the public interest in instances “injurious to public life, public health, and safety or the administration of justice”.^151^ Mance LJ dissented on the basis that the public interest could not be reduced to such “precise categorisations”.^152^ This dissent and the approach taken in *Lion Laboratories* was followed in *Ashdown v. Telegraph*, which reiterated that there are “those rare cases [where the public interest] trumps the rights conferred by the Copyright Act.”^153^

Although not clarified, one interpretation could be that the *Hyde Park* reference to public health relates to the injurious nature of the work placing it squarely within the public interest defence. However, narrowly read, the reference to public health might be less likely to apply to materials that merely improve public health. There is insufficient guidance to be sure. As the court noted in *Ashdown*, “It will be very rare for the public interest to justify the copying of the form of a work to which copyright attaches.”^154^ What is interesting to consider is the dynamic of enforcing copyright in works that improve public health during crises. Following *Ashdown*, it is now less likely that a court would put the public at a disadvantage when access barriers become injurious to the public(s) interest(s). However, overall, this piecemeal approach is inadequate to address the broader challenges raised during crises.

#### Morality and Ideas

Two relevant but thin approaches include morality and the idea/expression dichotomy. Although morality has been present in UK copyright law for over a century, it is rarely invoked.^155^ Discussions have intersected with the public interest, but the notion of morality appears to be restricted to subject-matter that would offend public sensibilities or prevent a breach of statutory duties, rather than more usefully, as a means to allow reproduction of works in times of crisis in the public(s) interest(s).^156^

The idea/expression dichotomy is another long-standing legal doctrine that protects the public domain and promotes independent creation in the public(s) interest(s). Copyright law’s refusal to protect ideas, unexpressed ideas, non-original elements, and expressions not associated with the work allows such material to remain in the public domain and be re-used. The complex nature of much copyright subject-matter means that the public must assess which elements of a work are not protected and remain available for re-use. This places a burden on the public, and the presence of copyright can chill re-use.

Courts make similar decisions when assessing elements of works.^157^ During a public health crisis, judicial discretion could be used to favour the withholding of copyright subsistence from works which could benefit public health. Even so, non-IPR and IPR barriers, like paywalls and DRMs, can be used to restrict access to elements ineligible for IPR protection and so this approach would be limited.

#### Non-Voluntary Licensing

Similar to patent law, non-voluntary licences can override copyright norms in specific and narrow circumstances.^158^ However, copyright schemes are underdeveloped and lack a unified approach.^159^ Relevant factors for the grant of a licence include exorbitant pricing, and the unmet needs of the public along with other producers and distributors.^160^ Non-voluntary licences have been criticised as “administratively cumbersome, unlikely to arrive at a correct rate, and contrary to copyright’s overall free market philosophy.”^161^ Unlike patent law, copyright law contains multiple permitted acts covering many circumstances in which a non-voluntary licence would be otherwise useful.

It is therefore unlikely that non-voluntary licences would be useful during a public health crisis in their current form. However, these schemes could be tailored more effectively to better enable their use when crises arise.

#### Restraint of Trade

The doctrine of restraint of trade was developed through case law to withhold the use of copyright in certain circumstances within the broader marketplace.^162^ Judges have applied the doctrine in a way that focuses on exclusive licence agreements between rightsholders and authors.^163^ Restraint of trade is often applied, but inconsistently, to rebalance the unequal bargaining positions of the contracting parties. 164 It is of limited use because the public(s) who seek access or use of IPR-protected content are rarely party to these agreements.

Restraint of trade would need to be reformulated to consider public(s) and access barriers. While this would require significant expansion, it is consistent with the spirit of the doctrine. Restraint of trade is often associated with the doctrine of illegality, which allows courts to set aside contracts that contravene the law or public policy.^165^ Courts have identified freedom from restraint of trade as one of such public policies.^166^ Judicial discretion could be used to interpret either doctrine in the public(s) interest(s) to enable access to health relevant IPR unfairly restrained by contractual terms as a matter of public policy.

#### Copyright Evaluation

There is an important procedural difference between copyright and patent law. Copyright arises automatically, whereas patents must be registered. Therefore, to test its limits, copyright must be enforced in court, which is why these doctrines have largely developed through case law.^167^ Consequently, this puts the public(s) seeking access at a disadvantage, as they must infringe copyright and invite a lawsuit to defend its use and test the boundaries of the rightsholder’s entitlements.

Additionally, their application is dependent upon varying degrees of judicial discretion resulting in inconsistency. If there is any context in which a broad interpretation of these doctrines would be justified, it would be a public health crisis. This may help resolve IPR access barriers, but cannot address the more prevalent non-IPR access barriers that are raised within this article. Existing public interest mechanisms in copyright law are therefore inadequate to reduce access barriers in a meaningful manner.

### Evaluation

Many of the pre-existing mechanisms discussed above are ineffective due to multiple factors, such as the procedural differences among types of IPR protection, the stage at which these doctrines are applied and the limited way in which the public(s) interest(s) are considered outside the legal boundaries of these doctrines. Some could be tailored more effectively or interpreted more broadly to provide additional access in times of crises, but non-IPR barriers persist. An overriding and unavoidable problem is that any national efforts will be territorially constricted. This fails the public(s) interest(s) during and outside public health crises. Directing attention to removing access barriers outside times of crises is therefore required to ensure more effective responses are in place when cross-border interventions become necessary. Overall, these pre-existing legal doctrines alone are insufficient to address access barriers in the public(s) interest(s) during a public health crisis.

## Conclusion

In considering the relationship between IPR and access in the “public interest”, this paper has examined the range of access barriers that are faced by various public(s), the methods used to grant access, and the effectiveness of these methods. We argue that neither open movements nor pre-existing legislative doctrines provide sufficient or effective solutions to the access barriers we have identified. Although significant advancements have been made through open movements that map onto IPR frameworks, movements that exclusively address IPR access barriers are inadequate. Moreover, traditional public interest mechanisms within existing IPR frameworks are so restricted and internally focused that they are ill-equipped to adapt to a crisis.

By contrast, the combination of existing non-voluntary mechanisms with open movements can go some way towards remedying issues relating to the voluntary nature of current open movements. This dual approach is the most promising to address the access barriers we have outlined within the confines of the current system, but it nonetheless fails to provide sufficient and effective solutions, and inequalities of access remain unresolved. The existing options are ineffective and insufficient because they fail to sufficiently address the wide range of existing non-IPR access barriers and public(s) interest(s). Binary distinctions and generalised framings that are common among these methods are inappropriate to reflect the diverse and plural public(s) interest(s) underpinning the welfare goals of IPR.

This problem is evident in the COVID-19 context. However, this crisis has only highlighted access barriers that have previously been an issue in many contexts, including global and national public health and in relation to educational and cultural heritage materials. The COVID-19 crisis has brought these issues to the attention of many who previously would not have been part of the public for whom access to IPR protected subject matter was problematic. What is clear is that the access barriers highlighted by COVID-19 and discussed in this paper are not restricted to narrow specific contexts but are widespread. They are created by, and are a feature of, our existing IPR frameworks. More room is required for a consideration of the public(s) interest(s), not just during a global health crisis, but on an ongoing basis. The “new normal” needs to advance equitable access to IPR protected subject matter for all public(s).

Accordingly, we propose that a systemic re-evaluation of IPR and access mechanisms is required to ensure public interest goals are (re)centred and shape both private and public responses to future crises. Part of this re-evaluation should include an examination of the effectiveness of the solutions put forward for addressing the public interest in this, and future, crises. The current pandemic and development of a “new normal” can provide a wake-up call to go further than the immediate pandemic and look to resolve the IPR issues that underlie access barriers. Otherwise, the scramble to remove these barriers during a public health crisis will continue to repeat itself, and inequalities will remain and deepen.

At present, IPR reform is a low priority for national governments and international organisations, even though access to IPR subject-matter is crucial to resolving the current pandemic and future public health crises. Countries have so far taken individual approaches to IPR reform that have largely been specific and focused on the COVID-19 public health context. By contrast, the Open COVID-19 initiative’s framework was designed for this crisis in a way that can be replicated and applied to future different global crises contexts, such as an environmental crisis. This is one reason why grassroots and industry IPR strategies developed: to fill gaps by reducing cross-border IPR access barriers. However, equitable long-term regulatory solutions that centre the public(s) interest(s) are essential to increase the likelihood that future crises will be less devastating and more efficiently and equitably resolved on a global level.

This requires international IPR legislation with defined and enforceable cross-border mechanisms to remove IPR and non-IPR access barriers that enters into force during crises. It must include clear and fair avenues for how “crisis” can be defined and adapted to new future crises. This should be created and developed through collaboration between governments, grassroots organisations, activists and representatives of all public(s). Particularly where public funding is involved, the public(s) should be normalised as interested parties in contracts, which should also include obligations to ensure the public(s) interest(s) are met. Of course, it is still imperative to develop national and international strategies. However, both national and international crises will require an international response due to the IPR and non-IPR barriers that arise. This is especially important for low/middle-income countries and communities.

The current pandemic, and development of a “new normal” provides a crucial moment to advance reform of the IPR system to better accommodate the public(s) interest(s). The willingness of governments, industry and policy-makers to work together towards the development of therapeutics and vaccines and to enable access to educational materials shows how much can be achieved in this way. Such efforts should be harnessed, so that reform goes further than the immediate crisis, and seeks to solve the problems outlined in this paper that underlie access barriers.

## Footnotes

1 Otero (2020); Brown, Ng and Adebola (2020); Stothers and Morgan (2020); Trimble (2020); “COVID-19 Special Issue” (2020) 42(9) EIPR; Contreras et al. (2020a); Gurry (2020).

2 The public welfare goals of IPR have been the subject of a rich body of literature. For one example, *see* Merges (2011).

3 *See* discussion below in section 3.

4 Here, “maker” is not used as a term of art or understood to carry meanings pertinent to specific industries (*e.g.* 3D printing) or regulations (*e.g.* Copyright Act 1968 (Australia), Sec. 22(3A)) referring to “makers of sound recordings” and not authors). It also captures the set of individuals who make new works that do not satisfy the threshold of originality as required by copyright, but nevertheless continue to claim rights in the outputs.

5 *See* Merges (2011). But, *see* Buccafusco and Sprigman (2019).

6 Pila (2017); Tushnet (2017).

7 Fisher and Oberholzer-Gee (2013). *See also* Barel and Boman (2020).

8 There have been some government initiatives to tackle national inflexibilities. In some countries, bespoke open government licences are used to release publicly-funded subject-matter. *See* “About Open Data” (National Palace Museum) https://theme.npm.edu.tw/opendata/Article.aspx?sNo=02009126&lang=2; and Loi n° 78-753 du 17 juillet 1978 portant diverses mesures d’amélioration des relations entre l’administration et le public et diverses dispositions d’ordre administratif, social et fiscal. The EU have adopted Creative Commons tools and licences to facilitate their own information sharing – Commission Decision of 22/02/2019 Adopting Creative Commons as an Open License under the European Commission’s Reuse Policy.

9 Discussed *infra* in section 3.2.

10 At the time of this writing, around 170 cultural heritage institutions, organisations and universities globally have stopped claiming copyright in faithful reproductions of public domain works as a matter of policy. *See* McCarthy and Wallace (2018).

11 A vast amount has been written on this topic and multiple organisations have been set up to tackle this issue. *See*, for example: Aritarathna and Kariyawasam (2020); Thambisetty (2019); Owoeye and Owoeye (2018); Manu (2014); Moon et al. (2012); ‘t Hoen et al. (2011). Organisations include but are not limited to: Unitaid, https://unitaid.org/; The Medicines Patent Pool, https://medicinespatentpool.org/: Gavi, https://www.gavi.org/; Knowledge Ecology International, https://www.keionline.org/; CEPI, https://cepi.net/; UNAIDS, https://www.unaids.org/en; Access to Medicine Foundation, https://accesstomedicinefoundation.org/.

12 In the copyright law context, the public(s) can be broken down even further according to the information sought. One public includes those who require access to copyright protected research, data, and other materials related to public health and the COVID-19 crisis. A second public includes those who are homebound, keeping connected via digital technologies, namely by moving to online-based education, 3D printing, and so on.

13 *See*, for example: Gana (1996); Rose (1996); Aoki (1998); Okediji (1999); Bartow (2006); Craig (2007); Craig (2014); Tushnet (2017), p. 113; Brauneis and Oliar (2018).

14 Strandburg (2018); von Hippel (2005); Craig (2011).

15 *See* Greenleaf and Lindsay (2018).

16 Unless a non-voluntary licence is granted by the government, as discussed below.

17 For more on this, *see* Price et al. (2020); Iqbal et al. (2020); Shadlen et al. (2013).

18 For a comprehensive overview, *see* UCL Institute for Innovation and Public Purpose (2018).

19 For example: the Bibliothèque nationale de France does not claim or charge a copyright fee, but restricts use to non-commercial purposes or requires a “user fee” (https://gallica.bnf.fr/edit/und/conditions-dutilisation-des-contenus-de-gallica); PACER (Public Access to Court Electronic Records) charges $0.10% to view United States federal judiciary opinions (https://pacer.uscourts.gov/). The terms and conditions of websites often claim rights in materials that extend beyond the reach of what copyright would recognise (Crews (2012); Wallace and Deazley (2016)). Also consider online streaming and protection under Sec. 18 CDPA 1988, as well as market access and competition aspects.

20 Vaver (2013), pp. 668–669. Supporting the framing of copyright exceptions and defences as users’ rights, *see CCH Canadian Ltd. v. Law Society of Upper Canada* [2004] SCC 13, para 48.

21 For patents, *see* Pogge, Rimmer and Rubenstein (2014); Drahos and Braithwaite (2002). For copyright, *see* Greenleaf and Lindsay (2018); Frosio (2012); Boyle (2008).

22 Chon (2006), pp. 2823–2824.

23 Chon critiques the concept of development in globalised intellectual property measures as a term of art with different contexts, applications and assumptions. *See ibid* 2818–20, 2823–25. *See also* Chander and Sunder (2004); Sunder (2006).

24 For more on this, *see* Bertoni (2012); Moon et al. (2012). Many organisations have been leading the way on providing access to medicines in low/middle-income countries and advocating for change. *See supra* note 11.

25 Over 60% of the world’s population remains offline according to a 2014 report, which also found 75% of that population is concentrated in 20 countries and is disproportionately rural, low income, elderly, illiterate, and female. Moreover, the lack of fixed-line infrastructure and high PC costs results in many users using their mobile devices to access the Internet. *See* McKinsey and Company (2014).

26 *See also* Burkett (2000).

27 “Joint Letter to Dr Francis Gurry” (2020); UNAIDS (2020).

28 *See* UCL Institute for Innovation and Public Purpose (2018); Mazzucato (2017); and ‘t Hoen (2020a).

29 The Wellcome Trust have been considering how to include access requirements for downstream inventions resulting from their funded research: “Wellcome’s Approach to Equitable Access to Healthcare Interventions” https://wellcome.ac.uk/what-we-do/our-work/access-healthcare-interventions/wellcomes-approach-equitable-access-healthcare-interventions. *See also* Hawkins, Garden and Winickoff (2021).

30 Licences are premised upon copyright and reserve some rights, whereas tools are used to dedicate materials to the public domain or signal their public domain status.

31 Kapsalis (2016), pp. 7–8.

32 “Principles and Implementation” https://www.coalition-s.org/addendum-to-the-coalition-s-guidance-on-the-implementation-of-plan-s/principles-and-implementation/. Signatories now include: the UK Research Institute, Wellcome Trust and the European Commission.

33 Kaye et al. (2009).

34 *See* Firefox, https://www.mozilla.org/en-US/mission/; Moodle, https://moodle.com/open-source/; Lego Ideas, https://ideas.lego.com/howitworks; NASA, https://www.nasa.gov/offices/oct/openinnovation/aboutus.

35 Van Overwalle (2015), p. 212, citing Chesbrough (2003). For similar, *see* Hope (2008); Van Overwalle (2009); Lee (2019); Chesbrough et al. (2013).

36 Van Overwalle (2015), p. 212, citing Chesbrough (2003).

37 Van Overwalle (2015), pp. 209–211.

38 Van Overwalle (2015), p. 220.

39 Van Overwalle (2015), p. 220.

40 Van Overwalle (2015), p. 210. For more on user innovation, *see* Strandburg (2018).

41 For more on the Medicines Patent Pool, *see* https://medicinespatentpool.org/.

42 For more on patent pledges and pools, *see* Aoki and Schiff (2008); Ebersole et al. (2005); Hope (2008); Van Overwalle (2009); Verbeure et al. (2006); Contreras and Jacob (2017).

43 Open software also falls under open access frameworks, as discussed below.

44 However, patenting software is controversial from both a “how to” protect and “whether to” protect perspective. For more, *see* Bakels and Hugenholtz (2002).

45 WHO (2020).

46 The MPP negotiates licences with pharmaceutical companies to provide affordable access to medicines for HIV/AIDS, tuberculosis and Hepatitis C in low/middle-income countries and has recently expanded its mandate to include COVID-19. *See* Unitaid (2020).

47 ‘t Hoen (2020b).

48 “About – Open Covid Pledge” https://opencovidpledge.org/about/. A call to join the pledge was published in Nature: Contreras et al. (2020b).

49 Neuburger (2020).

50 Contreras et al. (2020b).

51 *See* detailed information on licenses here: https://opencovidpledge.org/licenses/.

52 “OCL-PC v1.0 – Open COVID License 1.0” https://opencovidpledge.org/licenses/v1-0/.

53 For a list of these actions, *see* the WIPO COVID-19 IP Policy Tracker: https://www.wipo.int/covid19-policy-tracker/#/covid19-policy-tracker/ipo-operations.

54 That is, unless a compulsory licence is issued against them, as discussed further below.

55 DG Connect (2016); Leminen et al. (2012).

56 A list of pledgors is available at https://opencovidpledge.org/partners/. Pledged content includes designs for 3D printed respirators, swabs for testing, and techniques to aid in drug discovery. Further examples are available at https://opencovidpledge.org/partner-ip/. It will be interesting to note whether new inventions will be added to the pledge and whether all relevant inventions of those signing up have been pledged.

57 Fehrer (2020).

58 ‘t Hoen (2020c).

59 ‘t Hoen (2020a). *See also* ‘t Hoen and Boulet (2021).

60 Coronavirus Global Response, https://global-response.europa.eu/index_en.

61 WHO, “The Access to COVID-19 Tools (ACT) Accelerator”: https://www.who.int/initiatives/act-accelerator.

62 Gavi, “COVAX Facility”: https://www.gavi.org/covax-facility.

63 Boseley (2020). A separate but related point is the discrepancy between the cost of production and current price of remdesivir (as well as other medicines), highlighted in Hill et al. (2020).

64 Matthews (2020).

65 Fletcher et al. (2020); Callaway (2020).

66 “WHO Director-General’s opening remarks at the media briefing on COVID-19” (2021).

67 Communication from India and South Africa, “Waiver from certain provisions of the TRIPS Agreement for the prevention, containment and treatment of COVID-19” (2 October 2020) IP/C/W/669.

68 Mara (2020).

69 Suber (2012), p. 65.

70 Suber (2012), p. 65 – “digital, online, free of charge, and free of most copyright and licensing restrictions”.

71 Such as: Budapest Open Access Initiative, http://www.budapestopenaccessinitiative.org/; Bethesda Statement on Open Access Publishing, http://legacy.earlham.edu/%7Epeters/fos/bethesda.htm; Berlin Declaration, https://openaccess.mpg.de/Berlin-Declaration; Open Access Scholarly Publishers Association, https://oaspa.org/.

72 This includes: Public Domain Mark, CC0 1.0 Public Domain Dedication, CC BY, CC BY-SA, and equivalent statements (*e.g.* “no known copyright restrictions”).

73 Suber (2012), pp. 52–65.

74 Wellcome (2020a); “Oxford University Press – COVID-19”: https://global.oup.com/about/covid19?cc=gb.

75 The Copyright Licensing Agency in the UK temporarily revised the CLA Higher Education License, which expired 30 June 2020: “Addendum Amending CLA Higher Education Licence” https://cla.co.uk/sites/default/files/HE%20Licence%20Amendment%20Addendum.pdf. The UK’s sector-leading network of libraries (Research Libraries UK) requested the government to issue a statement allowing public libraries, research organisations and educational establishments to rely on the Sec. 171(3) CDPA for the period of crisis: “Letter to ministers” (2020).

76 “COVID-19 Open Access Letter” (2020).

77 Wellcome (2020b).

78 “Joint Letter to Dr Francis Gurry” (2020).

79 “Public Statement: Fair Use & Emergency Remote Teaching & Research” (2020). *See also* Hudson and Wragg (2020).

80 Lees (2020).

81 McCarthy and Wallace (2018).

82 Globally, almost 750 GLAMs, Universities and other organisations make some or all eligible data (*i.e.* images and collections data) available under open compliant reuse terms. This represents a small portion of cultural heritage organisations and institutions worldwide. *See* Wallace and Deazley (2016).

83 Hadley (2020), pp. 16–20.

84 Hadley (2020), p. 19.

85 “Museums around the World in the Face of COVID-19” (2020). *See* *also* “Museums, Museum Professionals and COVID-19” (2020); “Survey on the Impact of the COVID-19 Situation on Museums in Europe” (2020).

86 The National Museum in Prague. *See* McCarthy and Wallace (2018).

87 *See* Tanner (2004), p. 40; Dryden (2011); Kelly (2013), pp. 4–5; Pekel (2015), p. 9; Light (2015); Schmidt (2017), pp. 31–32; Valeonti et al. (2018), p. 57.

88 UNESCO Recommendation on Open Educational Resources (2019).

89 “#MuseumsFromHome” http://midatlanticmuseums.org/resources-museumsfromhome/.

90 It is worth noting materials submitted to social media and online platforms will be reusable by the public(s) according to the service providers’ terms and conditions.

91 Chakchouk and Giannini (2020).

92 “Fighting COVID-19 through digital innovation and transformation” (2020).

93 *See* Wallace and Euler (2020).

94 The text expressly ensures “the public can reliably and without fear of later claims of infringement build upon, modify, incorporate in other works, reuse and redistribute as freely as possible in any form whatsoever and for any purposes, including without limitation commercial purposes. These owners may contribute to the Commons to promote the ideal of a free culture and the further production of creative, cultural and scientific works, or to gain reputation or greater distribution for their Work in part through the use and efforts of others.” *See* CC0 1.0 Universal Legal Code, https://creativecommons.org/publicdomain/zero/1.0/legalcode.

95 For example, *see* Angelopoulos (2012).

96 Stinson et al. (2018).

97 *See* data collected monthly via Amazon’s Alexa service: https://www.alexa.com/topsites.

98 Cameron (2006).

99 Mazzone (2006).

100 Crissinger (2015).

101 *See* Taylor and Gibson (2017).

102 Chon (2006); Correa (2000); Okediji (2003).

103 Chander and Sunder (2004). “COVID-19: Human development on course to decline this year for the first time since 1990” (2020).

104 *See supra* note 95.

105 American Alliance of Museums (2020).

106 Digital divide is more evident than ever with GLAMs – 5% of museums in Africa and Small Island Developing States have been able to put content online. *See* “Museums around the World in the Face of COVID-19” (2020).

107 In fact, the temporary status may additionally benefit the maker post-crises.

108 WTO, “Intellectual Property and the public interest”.

109 For example, a patent will only be granted if it has been sufficiently disclosed. This requirement ensures that once the 20-year exclusive right comes to an end, the public will have the necessary information to make that invention without requiring any further research. Elaborated upon in: *Regeneron Pharmaceuticals Inc v. Kymab* [2020] UKSC 27, [23] and *Warner-Lambert v. Generics (UK) Ltd t/a Mylan* [2018] UKSC 56, [17].

110 Public interest was recently considered in *Evalve & Abbott v. Edwards Lifesciences* [2020] EWHC 513 (Pat).

111 *See* Pila (2017); and Tushnet (2017).

112 Art. 53(a) EPC. The corresponding provision of the UK Patents Act 1977 (PA), Sec. 1(3), uses the term “public policy” rather than “*ordre public*”, but the terms equate: Cole and Davis (2019), p. [1.21]. The Biotechnology Directive, implemented through Rule 28 EPC and Schedule A2 PA, sets out a number of specific cases where patenting is immoral.

113 T356/93 *Plant Genetic Systems/Glutamine Synthetase Inhibitors* of 21.2.1995. For more on *ordre public* and morality, *see* Walsh and Hawkins (2020).

114 T0866/01 *Euthanasia Compositions/Michigan State University* of 11.5.2005, [5.8].

115 T1213/05 *Breast and Ovarian Cancer/University of Utah* of 27.9.2007, [52]–[53].

116 Art. 53(c) EPC and Sec. 4A PA. This exception does not apply to products for use in these methods.

117 On *in vitro* testing, *see* Cole and Davis (2019), p. [4A.07]; and UK IPO, Examination Guidelines for Patent Applications relating to Medical Inventions in the UK Intellectual Property Office (UKIPO, Newport 2016) [56]. On genetic testing, *see* T0080/05 *Method of Diagnosis/University of Utah* of 19.11.2008, [62]–[63].

118 Sec. 60 PA, based on Art. 25 of the Community Patent Convention.

119 Sec. 60(5)(b) PA.

120 *Monsanto Co v. Stauffer Chemical Co* [1985] RPC 515 (CA) 542.

121 Cook (2006), pp. 133–134; Bor (2006).

122 Sec. 60(5)(c) PA. The corresponding provision of the Community Patent Convention is Art. 27(c).

123 Domeij (2000), p. 310.

124 Sec. 61 PA.

125 *Chiron Corporation v. Organon Teknika (No 10)* [1995] RPC 325.

126 *Edwards Lifesciences LLC v. Boston Scientific Scimed Inc* [2018] EWHC 1256 (Pat); *Evalve & Abbott v. Edwards Lifesciences Limited* [2020] EWHC 513 (Pat), [55]. For an international perspective, *see* Basheer et al. (2014).

127 Compulsory licensing is approved under TRIPS as long as it is compliant with Art. 31.

128 Sec. 48 and 48A PA.

129 Directive 2004/27/EC on the Community code relating to medicinal products for human use [2004] OJ L 136/34. For a more detailed analysis, *see* Li and Lim (2014).

130 For a more detailed discussion, *see* Aritarathna and Kariyawasam (2020), pp. 111–114; van Zimmeren and Van Overwalle (2011).

131 The application of Crown Use provisions (discussed following this section) was also argued.

132 “NHS England concludes wide-ranging deal for cystic fibrosis drugs” (2019).

133 ‘t Hoen (2020c).

134 For more on this work, *see supra* note 11.

135 For a list of these actions, *see* the WIPO COVID-19 IP Policy Tracker.

136 Germany – Act for the Protection of the Population in the Event of an Epidemic Situation of National Significance of 27 March 2020. France – Emergency Law No 2020-290 of 23 March 2020 to combat the COVID-19 epidemic. Chile has introduced legislation allowing compulsory licences on medicines, vaccines and technologies (https://www.keionline.org/chilean-covid-resolution).

137 Garrison (2020); Love (2020).

138 Sec. 55 and 56 PA. Many jurisdictions have similar government use provisions. The WIPO IP Policy tracker includes a list of existing legislative measures in all countries.

139 This is one of three examples in a non-exhaustive list.

140 Sec. 59 PA. To apply, an Order of Council declaring a period of emergency would be required.

141 *IP Com v. Vodafone* [2020] EWHC 132 (Pat).

142 *IP Com v. Vodafone* [2020] EWHC 132 (Pat), [207]–[213].

143 *Evalve & Abbott v. Edwards Lifesciences Limited* [2020] EWHC 513 (Pat), [77].

144 *See* Levine (2020).

145 Examples include temporary copying, reporting current events, disability access and parody (Secs. 28A, 30, 31A, 30A CDPA 1988 respectively).

146 Hudson and Wragg (2020).

147 Hudson and Wragg (2020).

148 Discussed immediately below.

149 For more on these cases, *see* Mysoor (2020), pp. 19–23.

150 *Lion Laboratories v. Evans* [1985] QB 526, 549.

151 *Hyde Park Residence Ltd v. Yelland and Others* [2000] EWCA Civ 37.

152 *Hyde Park Residence Ltd v. Yelland and Others,* [83].

153 *Ashdown v. Telegraph Group Ltd* [2002] RPC 5, [237].

154 *Ashdown v. Telegraph Group Ltd*, [251].

155 *See Glyn v. Weston Film Feature* [1916] 1 Ch 261; *AG v. Guardian Newspapers Ltd (No 2)* [1990] 1 AC 109.

156 *See Glyn v. Weston Film Feature*; *AG v. Guardian Newspapers Ltd (No 2)*.

157 *Kenrick & Co. v. Lawrence & Co.* (1890) 25 QBD 99; *Designers Guild v. Williams* [2000] UKHL 58, [6].

158 *See*, *inter alia* Secs. 63, 73, 135A–H, 144 CDPA 1988; Sec. 176, Sch. 17, Broadcasting Act 1990. Examples include: sound recordings in broadcasts and cable transmissions, orphan works, or duration. For a full list, *see* Caddick et al. (2016), Chapter 28 Part II.

159 The reason for this might be that the international rules to which the UK subscribes are not favourable to such schemes. Nonetheless, the Berne Convention does permit the granting of these sorts of licences.

160 Caddick et al. (2016).

161 Ginsburg (1990), p. 1924.

162 Restraint of trade is similar to the US copyright misuse doctrine, although the latter operates as a defence, not a cause of action in itself.

163 *See Schroeder Music Publishing v. Macaulay* [1974] 1 WLR 1308, 1314; Joined Cases *Silvertone Records v. Mountfield, Zomba Music v. Mountfield* [1993] EMLR 152, 160.

164 *Panayiotou and Others v. Sony Music Entertainment* [1994] EMLR 229, 345.

165 *Nordenfelt v. Maxim Nordenfelf Guns & Ammunition Co Ltd* [1984] AC 535 (HL); *Egon Zehnder Ltd v. Tillman* [2020] AC 154, 163–164.

166 *Egon Zehnder Ltd v. Tillman* [2019] UKSC 32; [2020] AC 154, 157.

167 The limits of patent law are also tested through litigation, but are first considered at the examination stage without the need for third party intervention.
